# 398. Increased Risk of Severe COVID-19 Outcomes Across all Groups of Individuals with Hematological Malignancies, Solid Tumors, and Solid Organ Transplants Compared with the General Population: Initial Results from INFORM, a Retrospective Health Database Observational Study in England

**DOI:** 10.1093/ofid/ofad500.468

**Published:** 2023-11-27

**Authors:** Richard McNulty, Sabada Dube, Yi Lu, Sophie Graham, Sofie Arnetorp, Nahila Justo, Renata Yokota, Kathryn Evans, Sudhir Venkatesan, Mark Yates, Sylvia Taylor, Jennifer Quint, Rachael A Evans

**Affiliations:** Medical Affairs, Vaccines and Immune Therapies Unit, AstraZeneca, Cambridge, UK, Cambridge, England, United Kingdom; Medical Evidence, Vaccines and Immune Therapies Unit, AstraZeneca, Cambridge, UK;, Cambridge, England, United Kingdom; Real-World Evidence, Data Analytics, Evidera, London, UK, London, England, United Kingdom; Real-World Evidence, Data Analytics, Evidera, London, UK, London, England, United Kingdom; Health Economics and Payer Evidence, BioPharmaceuticals R&D, AstraZeneca, Gothenburg, Sweden, Gothenburg, Vastra Gotaland, Sweden; Real-World Evidence, Data Analytics, Evidera, Stockholm, Sweden and Department of Neurobiology, Care Science and Society, Karolinska Institute, Stockholm, Sweden, Stockholm, Sodermanlands Lan, Sweden; P95, Leuven, Belgium, Dilbeek, Luxembourg, Belgium; Real-World Evidence, Data Analytics, Evidera, Waltham, MA, USA, Waltham, Massachusetts; Medical and Payer Evidence Statistics, BioPharmaceutical Medical, AstraZeneca, Cambridge, UK, Cambridge, England, United Kingdom; Real-World Evidence, Data Analytics, Evidera, London, UK, London, England, United Kingdom; Medical Evidence, Vaccines and Immune Therapies Unit, AstraZeneca, Cambridge, UK, Cambridge, England, United Kingdom; National Heart and Lung Institute, Imperial College London, London, UK, Cambridge, England, United Kingdom; University of Leicester, Leicester, England, United Kingdom

## Abstract

**Background:**

Despite COVID-19 vaccination, individuals who are immunocompromised due to underlying conditions, such as cancer and organ transplant, are at a greater risk of severe COVID-19 outcomes compared with the general population that is vaccinated. However, there are limited data quantifying this risk during the omicron-predominant period, particularly for important sub-groups that may have variable risk for severe COVID-19 outcomes. We report initial findings from the INFORM study describing the clinical burden and severe COVID-19 outcomes in patients with immunocompromised conditions (IC) in England, UK in 2022.

**Methods:**

This retrospective cohort study utilized National Health Service database in England. The study period was from Jan 1–Dec 31, 2022 and the baseline period for assessment of patient characteristics, including IC, was from Jan 1, 2017–Dec 31, 2021. Definitions of IC sub-groups are provided in **Table 1**. Severe COVID-19 outcomes were defined as COVID-19–related hospitalization, COVID-19–related intensive care unit (ICU) admission, and/or COVID-related death.

**Figure:**
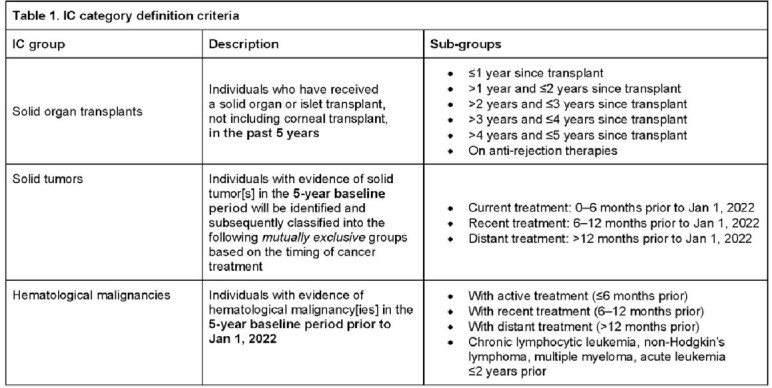

**Results:**

Of the 11,990,730 individuals in the general population sample aged ≥ 12 years, 468,745 (3.9%) were immunocompromised and accounted for approximately one-quarter of severe COVID-19–related outcomes (22% of hospitalizations, 28% of ICU admissions, and 23% of deaths; **Table 2**). The risk of severe COVID-19 outcomes was higher in well-recognized IC conditions (eg, solid organ transplants, hematological malignancies, and solid tumors) across all the sub-groups compared with the overall population (**Table 2**).

**Figure:**
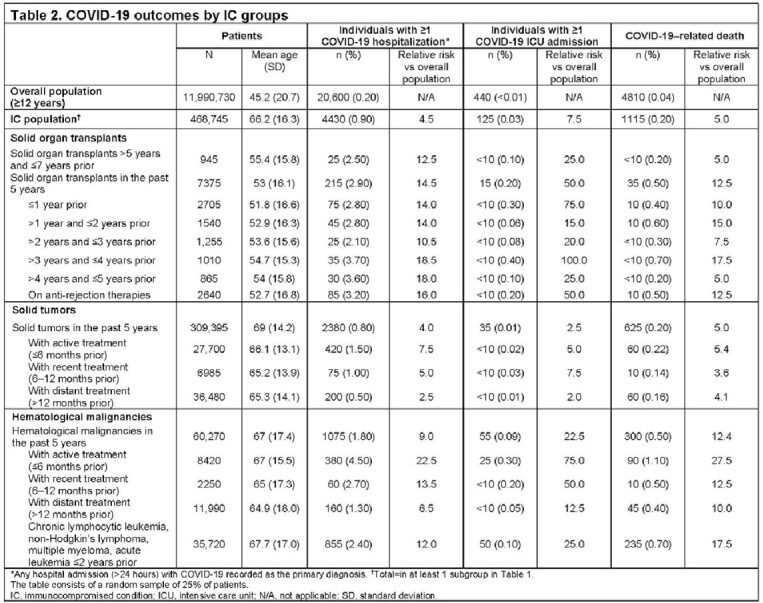

**Conclusion:**

There is an increased burden in the prevalence of severe COVID-19 outcomes among all individuals with IC, highlighting the need for additional protection to target this population.

**Disclosures:**

**Richard McNulty, MD**, AstraZeneca: Employee **Sabada Dube, PhD**, AstraZeneca: Employee **Yi Lu, PhD**, Evidera: Employee **Sophie Graham, MSc**, Evidera: Employee **Sofie Arnetorp, MS**, AstraZeneca: Employee **Nahila Justo, PhD, MBA**, Evidera: Employee|Karolinska Institute: Employee **Renata Yokota, PhD**, AstraZeneca: Employee **Kathryn Evans, MPH**, Evidera: Employee **Sudhir Venkatesan, MPH, PhD**, AstraZeneca: Employee **Mark Yates, PhD**, Evidera: Employee **Sylvia Taylor, PhD, MPH, MBA**, AstraZeneca: Stocks/Bonds **Jennifer Quint, PhD**, AstraZeneca: Grant/Research Support|Evidera: Grant/Research Support|GlaxoSmithKline: Grant/Research Support|Insmed: Grant/Research Support **Rachael A. Evans, PhD FRCP**, AstraZeneca: Advisor/Consultant|Boehringer: Advisor/Consultant|Evidera: Advisor/Consultant

